# Mediterranean Spotted Fever in Southeastern Romania

**DOI:** 10.1155/2013/395806

**Published:** 2013-08-18

**Authors:** Daniela Pitigoi, Ioana D. Olaru, Daniela Badescu, Alexandru Rafila, Victoria Arama, Adriana Hristea

**Affiliations:** ^1^National Institute of Infectious Diseases “Prof. Dr. Matei Bals”, 021105 Bucharest, Romania; ^2^Carol Davila University of Medicine and Pharmacy, 020022 Bucharest, Romania; ^3^National Institute of Research and Development for Microbiology and Immunology (Cantacuzino), 050096 Bucharest, Romania

## Abstract

Although cases of Mediterranean spotted fever (MSF) have been reported for decades in southeastern Romania, there are few published data. We retrospectively studied 339 patients, diagnosed with MSF at the National Institute of Infectious Diseases “Prof. Dr. Matei Bals” between 2000 and 2011, in order to raise awareness about MSF in certain regions of Romania. According to the Raoult diagnostic criteria 171 (50.4%) had a score >25 points. Mean age was 52.5 years. One hundred and fifty-five (90.6%) patients were from Bucharest and the surrounding region. Almost all patients presented with fever (99.4%) and rash (98.2%), and 57.9% had evidence of a tick bite. There were no recorded deaths. Serologic diagnosis was made by indirect immunofluorescence assay. Of the 171 patients, serology results for *R. conorii* were available in 147. One hundred and twenty-three (83.7%) of them had a titer IgG ≥1 : 160 or a fourfold increase in titer in paired samples. MSF is endemic in southeastern Romania and should be considered in patients with fever and rash even in the absence of recognized tick exposure. Since the disease is prevalent in areas highly frequented by tourists, travel-associated MSF should be suspected in patients with characteristic symptoms returning from the endemic area.

## 1. Introduction

Mediterranean spotted fever (MSF) also called boutonneuse fever is a tick-borne disease caused by *Rickettsia conorii* and transmitted to humans by the brown dog tick, *Rhipicephalus sanguineus*. MSF is traditionally considered to be endemic to the regions bordering the Mediterranean basin including southern Europe and northern Africa. Among the European countries with relatively high *R. conorii* infection rates are Portugal, Spain, France, and Italy [1–4]; however, the disease is also present in central and eastern Europe, central and southern Africa, and India [3]. It has also been reported in travellers to endemic regions returning to their native countries [5].

*R. conorii* infection has been described by most countries from the Balkan region. MSF cases have been reported in Bulgaria, Croatia, continental Greece, and the province of Thrace in Turkey [6–9], while serological evidence of infection was found in patients without MSF from Serbia [10] and ticks from Albania [11]. 

Cases of MSF have been reported in Romania since 1910, with the first described outbreak occurring in 1948 in the Bucharest area and in Dobrogea [12]. During the following years the incidence decreased, and after 1959, 1-2 cases per year were reported. Since 1988 small-sized outbreaks were described usually limited to members of the same family or community [13]. Since 2000, the National Institute of Public Health conducted a systematic surveillance of MSF [13]. MSF is endemic in southeastern Romania, with an overall incidence in 2009 of 0.7 per 100,000 population [14]. However, in some regions the incidence is much higher reaching 20 per 100,000 population per year. The majority of reported cases occur in 2 regions, Bucharest and the surrounding area and Dobrogea. A steady decrease in MSF incidence has been recorded during the last decade in Romania. A serological survey conducted in the MSF endemic area of southeastern Romania in 2009 detected high *R. conorii *IgG seroprevalence rates. The highest prevalence was recorded in Constanta county where specific antibodies were detected in almost a third of the individuals tested, followed by the Tulcea county and Bucharest with seropositivity frequency of 21.1% and 18.2%, respectively [15].

The objective was to raise awareness about MSF in certain regions of Romania, which is not a Mediterranean country, and to describe clinical and epidemiological characteristics in this area. 

## 2. Methods

### 2.1. Patient Population

Retrospective study of patients aged over 14 years, reported as having MSF according to epidemiological, clinical, and laboratory characteristics by the National Institute of Infectious Diseases “Prof. Dr. Matei Bals” between 2000 and 2011 was conducted. The hospital is a referral center for infectious diseases in Bucharest, where patients from half of the districts of Bucharest and six surrounding counties are treated. Patient records were analysed, and data on epidemiological, clinical, and laboratory features were collected. 

According to the diagnostic criteria described by Raoult et al., patients were further analysed if they had a diagnostic criterion score of >25 (Table 1) [16].

### 2.2. Serological Diagnosis

Paired serum samples collected at 10–14 days interval and single serum samples were tested by the indirect immunofluorescence assay method for anti-*R. conorii *IgG antibody levels. Serum sample dilutions were 1/40 and 1/80, and examination of the slides was made on Eurostar III Plus microscope at 400x magnification. The interpretation of the test results was determined by the presence of apple green fluorescence of cocobacilar morphology, the fluorescence pattern being the positive and negative controls provided by the kit producer (*Rickettsia conorii* IFA IgG kit produced by Vircell, Spain). Each positive serum by the screening test was analysed by twofold dilutions up to 1/640. The highest serum dilution with visible fluorescence (positive reaction) was considered the final titre of the serum.

Data were processed and analyzed by SPSS v17.0 (Statistical Package for the Social Sciences Inc, Chicago, IL, USA).

The study protocol was approved by the local ethics committee.

## 3. Results

Of 339 patients with reported MSF identified during the study period, 171 (50.4%) had a diagnostic score >25 points based on the Raoult criteria, and thus they were further analyzed. The mean age of patients was 52.5 years with a male to female sex ratio of 1 : 1.06. One hundred and fifty-five (90.6%) patients were from Bucharest and the surrounding region with the rest coming from other counties; 120 (70.2%) patients lived in an urban area. The number of cases per year ranged between 1 in 2006 and 42 in 2002 with 115 (67%) of cases diagnosed between 2000 and 2005. 

MSF cases were reported between May and November, predominantly during late summer months. Most cases were diagnosed in August (55, 32%) and July (40, 23%). 

Almost all patients presented with fever 170/171 (99.4%) and rash 168/171 (98.2%), but only 99/171 (57.9%) had evidence of a tick bite. Patient characteristics according to the Raoult et al. diagnosis criteria are shown in Table 1. 

Other common clinical symptoms encountered were headache in 66/153 (43.1%), myalgias in 66/152 (43.4%), arthralgias in 36/152 (23.7%), renal function impairment in 34/149 (22.8%), central nervous system symptoms in 7/149 (4.7%), and respiratory symptoms in 25/149 (16.8%) patients. Among patients with available laboratory tests, 54/170 (31.8%) had a white blood cell count >10,000/mm^3^, 81/159 (50.9%) had thrombocytopenia (platelet count < 150,000/mm^3^), 124/156 (79.5%) had an elevated erythrocyte sedimentation rate (>20 mm/h), 76/138 (55.1%) had increased plasma fibrinogen levels (>450 mg/dL), and 124/158 (78.5%) had elevated liver enzymes. Of the 171 patients, serology results for *R. conorii *were available for 147 (86%), and 123 of them (83.7%) had an IgG serum titer ≥1 : 160 or a fourfold increase. All serum samples with a positive screening test result at 1/80 dilution also tested positive at 1/160 dilution. There were no recorded deaths in our patient population.

## 4. Discussion

MSF is endemic in southeastern Romania with cases reported ever since the beginning of the twentieth century and evidence of past infection in up to a third of the tested individuals in some counties. The endemic area for MSF in Romania comprises regions which are highly frequented by tourists: Bucharest, the Danube Delta, and the coastal area bordering the Black Sea. This study describes the epidemiological, clinical, and laboratory characteristics of patients presenting to a large infectious diseases hospital from Bucharest, Romania.

In Romania, ticks and tick-borne diseases have been documented ever since the end of the 19th century. Up to now, 27 tick species are known to occur in Romania, 25 of them belonging to Ixodidae family (*Dermacentor marginatus; Dermacentor reticulatus; Haemaphysalis concinna; Haemaphysalis inermis; Haemaphysalis parva; Haemaphysalis punctata; Haemaphysalis sulcata; Hyalomma aegyptium; Hyalomma detritum scupense; Hyalomma marginatum marginatum; Ixodes apronophorus; Ixodes arboricola; Ixodes crenulatus; Ixodes hexagonus; Ixodes laguri; Ixodes redikorzevi; Ixodes ricinus; Ixodes rugicollis; Ixodes simplex; Ixodes trianguliceps; Ixodes vespertilionis; Rhipicephalus annulatus; Rhipicephalus bursa; Rhipicephalus rossicus; Rhipicephalus sanguineus*) and only two to *Argasidae* family (*Argas persicus* and *Argas reflexus*) [17]. 

The studies show that in Romania, *Rh. sanguineus* begins the questing activity in April and remains active throughout the warm season until November [17]. The hosts for *Rh. sanguineus* are the following: human; companion animals; livestock; rodents.

At the national level, the highest incidence (44 cases per 100,000 population) was recorded in 2001 in Constanta county, whereas in Bucharest the number of MSF cases peaked in 2002 (3.15 cases per 100,000 population). After this peak that occurred a decade ago, the incidence decreased in all endemic areas of Romania [13]. A similar epidemiological trend was recorded in neighbouring Bulgaria [6]. Other authors described fluctuating trends of MSF incidence in Spain, Italy, France, and Croatia [3, 7]. 

We also found in our study a decreasing trend in the number of cases presenting at our hospital with more than two-thirds of the cases diagnosed during the first half of the study period. It should be noted that because of reduced disease severity not requiring hospital admission and the inability to perform serology in all suspected cases, MSF might be underdiagnosed and underreported. Most cases were identified during late spring and summer, which is the period of greatest tick activity. The peak in the number of cases in August might be attributed to the preponderant involvement of immature tick stages in disease transmission [18]. We found that the majority of patients diagnosed with MSF were living in an urban area.

Since dogs are the main hosts for *Rh. sanguineus,* individuals with frequent contact with dogs are at increased risk for developing the disease [19, 20]. We hypothesize that the stray dogs in Bucharest might have contributed to a greater likelihood of contact between humans and the vector.

The classical clinical picture of MSF is comprised of fever, rash, and the presence of a black eschar. Fever and rash were seen in almost all patients, and the eschar was also commonly reported in over 67% of individuals. Although *R. conorii *is the main cause of MSF in Europe, other rickettsial species (such as *R. monacensis*, *R. massiliae, *and *R. aeschlimannii*) have been shown to produce a MSF-like disease [21]; however, data on other *Rickettsia spp. *that may cause a similar disease in Romania are lacking. MSF mortality is generally low, estimated at about 2.5% [18]. In our study none of the patients died, but severe cases might have been underdiagnosed because MSF may not have been considered as a diagnosis in critical patients with rash or because serological tests may have been negative if performed too early in the course of the disease. According to the national MSF surveillance data between 2000 and 2008, only 2 deaths were reported [13]. Other studies from the region also found low mortality rates [6, 22]. Some *R. conorii *strains, however, have been reported to cause higher mortality rates, over 32% in hospitalized patients [1]. 

Although most of the patients had serological evidence of MSF, in some cases there was a delay of 7–15 days in antibody detection after disease onset [18], which might explain an initial negative serology. Another possible explanation for a negative serology could be infection by another rickettsial species causing MSF-like disease [21].

In order to improve surveillance of MSF, in 2012, the National Institute of Public Health implemented a new surveillance methodology. The case definition was based on epidemiological criteria (tick bite or exposure, contact with dogs, professional exposure, or recreational activities in potentially tick-infested areas), clinical criteria (fever, myalgias, nonpruritic, or maculopapular rash predominantly affecting limbs with/without the presence of a 2–5 mm lesion suggesting a tick bite), and laboratory criteria (increase in specific IgM antibody levels in paired serum samples or fourfold increase of specific IgG levels in paired serum samples). A case of MSF was considered confirmed if epidemiological, clinical, and laboratory criteria were present. Patients are considered to have probable MSF if the clinical and epidemiological criteria were present and suspected when only the clinical criteria were fulfilled. The initiative of the National Institute of Public Health to implement a specific methodology might provide a more accurate picture of MSF epidemiology in Romania and increase awareness of the disease among general practitioners and therefore increased MSF reporting.

The limitation of our study was the retrospective analysis, which might have contributed to missing data. Furthermore, PCR techniques may have been a more useful and sensitive test to identify the *R. conorii *strain involved. We were also unable to identify other rickettsial species causing MSF-like disease in our patient population.

In conclusion MSF is endemic in southeastern Romania and should be considered in patients presenting with fever and rash even in the absence of recognized tick exposure. Since the disease is prevalent in areas highly frequented by tourists (Bucharest, the Danube Delta, and the coastal region) and there are increased chances of contact with stray dogs in these areas, travel-associated MSF should be considered in patients with characteristic symptoms returning from this endemic area.

## Figures and Tables

**Table 1:** Diagnostic criteria of Raoult et al. in the study patients.

	Points	Patient characteristics *N* (%)	Patients evaluated
*Epidemiological criteria*			
Stay in endemic area	2	171 (100)	171
Occurrence between May and September	2	165 (96.5)	171
Contact with dog ticks	2	114 (79.2)	144
*Clinical criteria *			
Fever > 39°C	5	170 (99.4)	171
Eschar	5	115 (67.3)	171
Maculopapular or purpuric rash	5	168 (98.2)	171
Two of the three clinical criteria	3	76 (44.4)	171
All three clinical criteria present	5	95 (55.6)	171
*Nonspecific laboratory findings *			
Platelets < 150,000/mm^3^	1	81 (50.9)	159
ALT or AST > 50 u/L	1	124 (78.5)	158
*Serological criteria *			
Sole serum sample IgG ≥ 1 : 160	10	92 (62.6)	147
Fourfold increase in paired serum samples	20	31 (21.1)	147
